# Annotations

**Published:** 1889-09-21

**Authors:** 


### Annotations.

**Asylums or Hospitals?** The proposals to establish hospitals for the insane by the London County Council seems to have caused much sensation in the asylum world. Many superintendents have referred to it in their annual reports, and among others we notice the following paragraph in the report of the Royal Asylum at Perth: "A few weeks ago the London County Council made a movement towards the establishment of an hospital for the insane on the lines of those general hospitals that have been an outstanding feature of modern civilisation. It is proposed that it shall be a place for the treatment of acute cases of insanity and the study of the problems involved—that it shall be, in fact, an effort to gain a greater proportion of recoveries than the world has yet known. The advantages and disadvantages of such an establishment might be debated at great length. It is a question whether the aggregation of so many acute cases, without the quiet, chronic patients who give a stability to the asylum microcosm, will be so beneficial as is hoped. The phalanx of failures, whose well-considered routine constitutes the *force majeur* of an ordinary asylum, and into whose orderly ways the newcomer of ill-regulated brain drops by sheer force of superior numbers—the chronic patients may be missed. Assuming that the difficulties of site and extent of grounds be overcome, so that these essentials of proper treatment—outdoor occupation and amusement—are duly secured; assuming that the costly working expenses be met; the legal responsibilities of the superintendent transferred to visiting physicians—granting that this plan is to arise phœnix-like from its discredited ashes, and to attain a success in the future that it never knew in the past, however it may succeed in the vast and wealthy metropolis, it is not adapted to the needs of such a locality as this. But it is very evident that it is for such institutions as this to adopt the principle in a modified form, to develop the medical idea, and to treat with individualisation acute cases in special wards." So writes Dr. Urquarht, and we have long since expressed similar views. Whether the metropolis will consent to try the experiment again remains to be seen.

**Dogs and Muzzles.** The muzzling order has been extensively obeyed, and we may hope to see rabies stamped out in the course of a twelvemonth. Many persons are unhappy on account of their canine friends, and contemplate the prospective year of durance with anything but gratification. A little pamphlet, published by Mr. W. Ridgway, of Piccadilly, and written by Mr. Alfred Cawdle, M.R.C.V.S., is well calculated to set the minds of lovers of dogs at rest on this point. In the first place, Mr. Cawdle assures his readers that nothing whatever except the prolonged muzzling of dogs will effectually stamp out rabies. The muzzle must be used for at least twelve months. Many people consider the police regulations arbitrary in themselves, and cruel to the dogs. Mr. Cawdle says "No." "I can assure them," he writes, "that a properly adjusted muzzle causes no pain, and in the majority of instances no inconvenience to the animal." Otherwise is it possible to imagine that "so intelligent a creature as the dog would come to his owner, and stand perfectly quiet until it is put on?" "Furthermore," he continues, "I have found that those dogs which are most opposed to it at first, no doubt merely because it is strange, get thoroughly accustomed to it after they have worn it two or three times." Even if muzzling were a permanent grievance to dogs, which it certainly is not, Mr. Cawdle thinks it very proper that their feelings should be sacrificed rather than that a near relation or even a stranger should contract so dreadful a disease as hydrophobia. Therein most persons whose opinions are entitled to consideration, will undoubtedly agree with him. In discussing the treatment of dogs which are certainly suffering from rabies, Mr. Cawdle is very decided in his opinions. "The only applicable remedy is to kill." "Kill the dog," he says, "directly he is known to be rabid. He will thereby be saved a great deal of pain himself, and all risk of inoculating other dogs or human beings is, of course, done away with." In the case of a human being bitten by a mad dog, Mr. Cawdle thinks that cauterisation is of great service, provided two conditions are borne in mind—viz., that it be done immediately and done effectually. In large towns there can seldom be any difficulty in carrying out both these conditions. Doctors or chemists may be found in every street, and if one of them should happen to be too timid or sensitive to cauterise sufficiently there is nothing to prevent the bitten person himself pushing the caustic stick into the wound and keeping it there for ten or fifteen minutes if necessary. The pain is very slight. Dog lovers in the country should carry about a small stick of caustic in their pockets, and thus be provided against all emergencies. Mr. Cawdle's pamphlet is small but practical, and only costs sixpence.

**Atraperna's Neighbour.** It has lately come to public knowledge that ether drinking is common in North of Ireland, and various schemes are talked of for suppressing the vice. But it must be obvious that to do this effectively the support and assistance of the chemist who sells the ether must be obtained. Restrictions may be laid on the sale of it, but it is all but impossible to enforce these unless the chemist's conscience bids him help the law. The chemist is a man of education, he has passed examinations that demand a fair amount both of scientific knowledge and of general culture, he holds a responsible and honourable position with regard to society. We are, therefore, justified in expecting from him something more than the selfish calculations of the common publican. What do we find? A correspondent of the *British and Colonial Druggist*, who signs himself "Atraperna," and writes from Dublin, thus states what he considers to be his duty in this matter of ether drinking, which, moreover, he designates an "abominable practice, which is filling the asylums of the North": "I was a considerable period in a Londonderry establishment, and we had a large number of customers for the oxide of ethyl. I was perfectly cognisant of the fact that the ether was for use as a beverage, but as there were no restrictions on its sale I could see no reason why I should take any philanthropic interest in other people's business and send them to the rival medical hall over the way." "Atraperna is very much of Cain's opinion with regard to being his brother's keeper. He sees people giving way to an "abominable practice," but it is no business of his to prevent it. His business in life is to prevent money going into the till of "the rival medical hall over the way." To him the ether drinker is not a neighbour to be treated with honesty and justice; he is not a brother to be saved from a degrading vice; he is only a customer, out of whom to squeeze a profit. It would be interesting to know what creed "Atraperna" professes, by what rules he professes to guide his conduct before God and man. Does he himself suspect that he is only a mammon worshipper, or does he think he is a Christian? One thing we know of him that he is not a happy man, and probably not a successful one; for he writes on two other subjects to the same journal, and in both his cry is one of complaint. He is angered, in the first place, that a Scotchman has received an appointment in Dublin. "Why are we Irish boycotted by employers in our own country?" Well, perhaps the employers have their own reasons, and these good enough. Again, he protests against the idea that there is any reason for the pharmacist being better educated than he is now. "Protection, not education, is wanted by the chemist," he says. Protection in the manufacture of ether-drunkards among other things, doubtless. The public also needs protection; even the drug-drunkard, who knows only the immediate exhilaration of his dram and not its far-reaching evil, needs to be protected from his own weakness. And we certainly think that the chemist, if we take "Atraperna" as a sample one—which, however, we decline to do—still needs a good deal of education, not perhaps in Latin and materia medica, but in his duty towards his neighbour.

